# Trimethyl­phenyl­ammonium μ-bromido-bis­[dibromidobis(4-bromobenzyl)stannate(IV)]

**DOI:** 10.1107/S1600536811021295

**Published:** 2011-06-11

**Authors:** Thy Chun Keng, Kong Mun Lo, Seik Weng Ng

**Affiliations:** a Department of Chemistry, University of Malaya, 50603 Kuala Lumpur, Malaysia

## Abstract

In the title salt, [C_6_H_5_N(CH_3_)_3_][Sn_2_Br_5_(C_7_H_6_Br)_4_], the Sn^IV^ atoms of the dinuclear anion are bridged by a Br atom; the Sn—Br_bridge_ bond lengths are 2.9818 (5) and 3.0470 (5) Å. Both Sn atoms show a distorted *cis*-trigonal–bipyramidal coordination geometry that is distorted towards a square pyramid. In the crystal, weak C—H⋯π inter­actions occur between anions and cations.

## Related literature

For the ferrocenium salt, see: Razak *et al.* (1998 ▶).
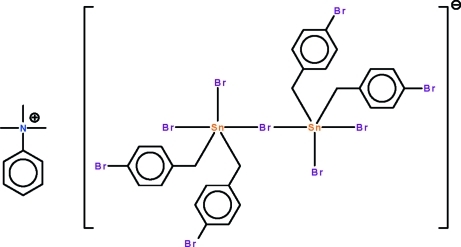


## Experimental

### 

#### Crystal data

(C_9_H_14_N)[Sn_2_Br_5_(C_7_H_6_Br)_4_]
*M* *_r_* = 1453.25
Monoclinic, 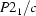

*a* = 12.7274 (2) Å
*b* = 25.5126 (3) Å
*c* = 13.5135 (2) Å
β = 100.8540 (7)°
*V* = 4309.46 (11) Å^3^
*Z* = 4
Mo *K*α radiation
μ = 9.53 mm^−1^
*T* = 100 K
0.25 × 0.20 × 0.20 mm

#### Data collection

Bruker SMART APEX diffractometer
Absorption correction: multi-scan (*SADABS*; Sheldrick, 1996 ▶) *T*_min_ = 0.369, *T*_max_ = 1.000
39991 measured reflections
9908 independent reflections
7661 reflections with *I* > 2σ(*I*)
*R*_int_ = 0.042

#### Refinement

*R*[*F* ^2^ > 2σ(*F* ^2^)] = 0.035
*wR*(*F* ^2^) = 0.075
*S* = 1.01
9908 reflections
442 parameters
H-atom parameters constrained
Δρ_max_ = 1.91 e Å^−3^
Δρ_min_ = −0.57 e Å^−3^

### 

Data collection: *APEX2* (Bruker, 2009 ▶); cell refinement: *SAINT* (Bruker, 2009 ▶); data reduction: *SAINT*; program(s) used to solve structure: *SHELXS97* (Sheldrick, 2008 ▶); program(s) used to refine structure: *SHELXL97* (Sheldrick, 2008 ▶); molecular graphics: *X-SEED* (Barbour, 2001 ▶); software used to prepare material for publication: *publCIF* (Westrip, 2010 ▶).

## Supplementary Material

Crystal structure: contains datablock(s) global, I. DOI: 10.1107/S1600536811021295/xu5234sup1.cif

Structure factors: contains datablock(s) I. DOI: 10.1107/S1600536811021295/xu5234Isup2.hkl

Additional supplementary materials:  crystallographic information; 3D view; checkCIF report

## Figures and Tables

**Table:** 

Sn1—C1	2.163 (4)
Sn1—C8	2.167 (4)
Sn1—Br1	2.6203 (5)
Sn1—Br2	2.5051 (5)
Sn1—Br6	3.0470 (5)
Sn2—C15	2.165 (4)
Sn2—C22	2.160 (4)
Sn2—Br6	2.9818 (5)
Sn2—Br5	2.5057 (5)
Sn2—Br7	2.6423 (5)

**Table:** 

C1—Sn1—C8	141.50 (16)
C15—Sn2—C22	138.41 (16)

**Table 2:** Hydrogen-bond geometry (Å, °) *Cg* is the centroid of the C29-benzene ring.

*D*—H⋯*A*	*D*—H	H⋯*A*	*D*⋯*A*	*D*—H⋯*A*
C27—H27⋯*Cg*^i^	0.95	2.58	3.378 (4)	142

Symmetry code: (i) 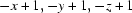.

**Fig. 1.**
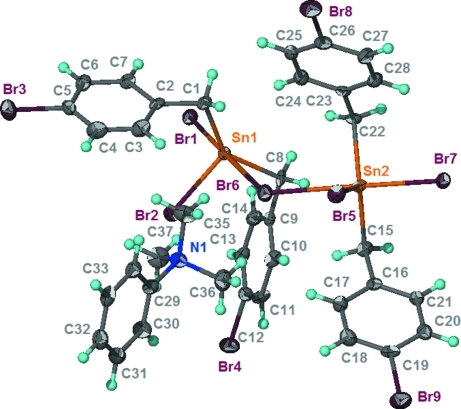
Thermal ellipsoid plot (Barbour, 2001) of C6H5N(CH3)3+ [Sn2Br5(C7H6Br)4]- at the 70% probability level; hydrogen atoms are drawn as spheres of arbitrary radius.

## supplementary crystallographic information

### Comment

We have reported the products of the reaction of phenyltrimethylammonium tribromide with triorganotin halides. The products are bis(ammonium) hexahalogenostannates as all tin-carbon bonds are cleaved. When heating was avoided, only one organic radical is cleaved, as found for the reaction of tris(*p*-bromobenzyl)tin bromide. In the salt, C_6_H_5_N(CH_3_)_3_^+^ [Sn_2_Br_5_(C_7_H_6_Br)_4_]^-^ (Scheme I), the Sn^IV^ atoms of the dinuclear anion is bridged by a Br atom. The Sn atom shows *cis*-trigonal bipyramidal coordination but the geometry is distorted towards a square pyramid (Fig. 1).

The [Sn_2_Br_5_(C_7_H_6_Br)_4_]^-^ anion is known only in a report on the ferrocenium derivative (Razak *et al.*, 1998).

### Experimental

Tris(*p*-bromobenzyl)tin bromide (0.35 g,0.5 mmol) and phenyltrimethylammonium tribromide (0.20 g, 0.6 mmol) were dissolved in a 1:1 mixture of chloroform and ethanol (100 ml). The mixture was stirred at room temperature for half an hour, after which it was filtered. The filtrate when allowed to evaporate yielded yellow crystals.

### Refinement

Carbon-bound H-atoms were placed in calculated positions (C—H 0.95 to 0.99 Å) and were included in the refinement in the riding model approximation, with *U*(H) set to 1.2 to 1.5*U*(C).

The final difference Fourier map had a peak in the vicinity of Sn2.

### Crystal data

**Table:**

(C_9_H_14_N)[Sn_2_Br_5_(C_7_H_6_Br)_4_]	*F*(000) = 2728
*M**_r_* = 1453.25	*D*_x_ = 2.240 Mg m^−^^3^
Monoclinic, *P*2_1_/*c*	Mo *K*α radiation, λ = 0.71073 Å
Hall symbol: -P 2ybc	Cell parameters from 9891 reflections
*a* = 12.7274 (2) Å	θ = 2.2–28.3°
*b* = 25.5126 (3) Å	µ = 9.53 mm^−^^1^
*c* = 13.5135 (2) Å	*T* = 100 K
β = 100.8540 (7)°	Block, yellow
*V* = 4309.46 (11) Å^3^	0.25 × 0.20 × 0.20 mm
*Z* = 4	

### Data collection

**Table:**

Bruker SMART APEX diffractometer	9908 independent reflections
Radiation source: fine-focus sealed tube	7661 reflections with *I* > 2σ(*I*)
graphite	*R*_int_ = 0.042
ω scans	θ_max_ = 27.5°, θ_min_ = 1.7°
Absorption correction: multi-scan (*SADABS*; Sheldrick, 1996)	*h* = −16→16
*T*_min_ = 0.369, *T*_max_ = 1.000	*k* = −33→33
39991 measured reflections	*l* = −17→17

### Refinement

**Table:**

Refinement on *F*^2^	Primary atom site location: structure-invariant direct methods
Least-squares matrix: full	Secondary atom site location: difference Fourier map
*R*[*F*^2^ > 2σ(*F*^2^)] = 0.035	Hydrogen site location: inferred from neighbouring sites
*wR*(*F*^2^) = 0.075	H-atom parameters constrained
*S* = 1.01	*w* = 1/[σ^2^(*F*_o_^2^) + (0.0341*P*)^2^ + 7.3533*P*] where *P* = (*F*_o_^2^ + 2*F*_c_^2^)/3
9908 reflections	(Δ/σ)_max_ = 0.001
442 parameters	Δρ_max_ = 1.91 e Å^−^^3^
0 restraints	Δρ_min_ = −0.57 e Å^−^^3^

### Fractional atomic coordinates and isotropic or equivalent isotropic displacement parameters (Å^2^)

**Table:**

	*x*	*y*	*z*	*U*_iso_*/*U*_eq_	
Sn1	0.81786 (2)	0.337729 (12)	0.94404 (2)	0.01448 (7)	
Sn2	0.70478 (2)	0.337048 (12)	0.54752 (2)	0.01443 (7)	
Br1	0.86566 (3)	0.287857 (17)	1.11616 (3)	0.01834 (10)	
Br2	0.92320 (3)	0.418920 (17)	0.99980 (3)	0.01920 (10)	
Br3	0.61807 (4)	0.52309 (2)	1.27020 (4)	0.03240 (13)	
Br4	1.38301 (4)	0.36296 (2)	0.97869 (4)	0.02558 (11)	
Br5	0.59378 (4)	0.416124 (18)	0.48842 (3)	0.02092 (10)	
Br6	0.75532 (4)	0.394348 (18)	0.74303 (3)	0.02003 (10)	
Br7	0.66142 (3)	0.287356 (18)	0.37302 (3)	0.01896 (10)	
Br8	0.14869 (4)	0.36492 (2)	0.56486 (4)	0.02844 (12)	
Br9	0.93742 (4)	0.516322 (19)	0.22308 (4)	0.02822 (12)	
N1	0.7352 (3)	0.56483 (15)	0.7507 (3)	0.0189 (8)	
C1	0.6533 (3)	0.35253 (18)	0.9558 (3)	0.0170 (9)	
H1A	0.6132	0.3644	0.8897	0.020*	
H1B	0.6203	0.3195	0.9734	0.020*	
C2	0.6447 (3)	0.39277 (17)	1.0331 (3)	0.0148 (9)	
C3	0.6342 (4)	0.44532 (19)	1.0054 (4)	0.0232 (10)	
H3	0.6324	0.4548	0.9372	0.028*	
C4	0.6263 (4)	0.48384 (19)	1.0753 (4)	0.0261 (11)	
H4	0.6185	0.5196	1.0555	0.031*	
C5	0.6299 (3)	0.46979 (18)	1.1740 (4)	0.0207 (10)	
C6	0.6394 (3)	0.41858 (18)	1.2051 (3)	0.0192 (9)	
H6	0.6411	0.4096	1.2736	0.023*	
C7	0.6466 (3)	0.38005 (17)	1.1337 (3)	0.0171 (9)	
H7	0.6530	0.3444	1.1540	0.021*	
C8	0.9143 (3)	0.28879 (17)	0.8651 (3)	0.0167 (9)	
H8A	0.9082	0.2517	0.8848	0.020*	
H8B	0.8882	0.2917	0.7915	0.020*	
C9	1.0284 (3)	0.30581 (17)	0.8906 (3)	0.0151 (9)	
C10	1.0675 (3)	0.34458 (18)	0.8336 (3)	0.0185 (9)	
H10	1.0218	0.3592	0.7765	0.022*	
C11	1.1727 (3)	0.36190 (18)	0.8598 (3)	0.0195 (9)	
H11	1.1991	0.3883	0.8213	0.023*	
C12	1.2381 (3)	0.34023 (17)	0.9423 (3)	0.0176 (9)	
C13	1.2024 (4)	0.30160 (18)	0.9994 (3)	0.0196 (10)	
H13	1.2489	0.2868	1.0557	0.024*	
C14	1.0973 (4)	0.28488 (17)	0.9725 (3)	0.0189 (9)	
H14	1.0718	0.2584	1.0114	0.023*	
C15	0.8704 (3)	0.35093 (18)	0.5378 (3)	0.0181 (9)	
H15A	0.9096	0.3633	0.6040	0.022*	
H15B	0.9033	0.3176	0.5220	0.022*	
C16	0.8814 (3)	0.39068 (18)	0.4590 (3)	0.0169 (9)	
C17	0.8833 (4)	0.44396 (19)	0.4823 (4)	0.0253 (11)	
H17	0.8750	0.4548	0.5477	0.030*	
C18	0.8968 (4)	0.48114 (19)	0.4118 (4)	0.0260 (11)	
H18	0.8974	0.5174	0.4280	0.031*	
C19	0.9096 (3)	0.46504 (18)	0.3176 (3)	0.0195 (9)	
C20	0.9056 (3)	0.41296 (17)	0.2910 (3)	0.0188 (9)	
H20	0.9126	0.4027	0.2250	0.023*	
C21	0.8913 (3)	0.37575 (17)	0.3617 (3)	0.0160 (9)	
H21	0.8881	0.3397	0.3439	0.019*	
C22	0.6133 (3)	0.28391 (16)	0.6225 (3)	0.0147 (9)	
H22A	0.6149	0.2484	0.5934	0.018*	
H22B	0.6456	0.2820	0.6950	0.018*	
C23	0.5001 (3)	0.30229 (16)	0.6105 (3)	0.0148 (9)	
C24	0.4648 (4)	0.32903 (18)	0.6869 (3)	0.0204 (10)	
H24	0.5127	0.3350	0.7488	0.025*	
C25	0.3601 (4)	0.34747 (18)	0.6745 (3)	0.0211 (10)	
H25	0.3359	0.3649	0.7283	0.025*	
C26	0.2930 (3)	0.34026 (18)	0.5848 (3)	0.0188 (9)	
C27	0.3249 (3)	0.31357 (19)	0.5063 (3)	0.0204 (10)	
H27	0.2768	0.3082	0.4443	0.025*	
C28	0.4287 (3)	0.29492 (18)	0.5205 (3)	0.0189 (9)	
H28	0.4517	0.2766	0.4673	0.023*	
C29	0.7368 (3)	0.62369 (18)	0.7491 (3)	0.0181 (9)	
C30	0.8351 (4)	0.64841 (18)	0.7540 (3)	0.0223 (10)	
H30	0.8986	0.6282	0.7594	0.027*	
C31	0.8400 (4)	0.70240 (19)	0.7508 (3)	0.0229 (10)	
H31	0.9070	0.7193	0.7534	0.027*	
C32	0.7478 (4)	0.7321 (2)	0.7439 (3)	0.0246 (10)	
H32	0.7512	0.7692	0.7408	0.030*	
C33	0.6507 (4)	0.70722 (19)	0.7416 (3)	0.0231 (10)	
H33	0.5878	0.7276	0.7386	0.028*	
C34	0.6438 (4)	0.65296 (19)	0.7435 (3)	0.0207 (10)	
H34	0.5768	0.6361	0.7410	0.025*	
C35	0.6252 (4)	0.54242 (19)	0.7476 (4)	0.0286 (11)	
H35A	0.5770	0.5548	0.6869	0.043*	
H35B	0.5982	0.5538	0.8074	0.043*	
H35C	0.6289	0.5041	0.7464	0.043*	
C36	0.7741 (4)	0.5443 (2)	0.6599 (4)	0.0306 (12)	
H36A	0.7283	0.5578	0.5986	0.046*	
H36B	0.7714	0.5060	0.6597	0.046*	
H36C	0.8479	0.5559	0.6620	0.046*	
C37	0.8053 (4)	0.5446 (2)	0.8456 (4)	0.0284 (11)	
H37A	0.7794	0.5582	0.9045	0.043*	
H37B	0.8791	0.5563	0.8480	0.043*	
H37C	0.8030	0.5062	0.8461	0.043*	

### Atomic displacement parameters (Å^2^)

**Table:**

	*U*^11^	*U*^22^	*U*^33^	*U*^12^	*U*^13^	*U*^23^
Sn1	0.01219 (14)	0.01765 (16)	0.01408 (14)	−0.00085 (11)	0.00372 (11)	−0.00305 (12)
Sn2	0.01197 (14)	0.01826 (16)	0.01353 (14)	0.00009 (11)	0.00359 (11)	0.00278 (12)
Br1	0.0195 (2)	0.0207 (2)	0.0148 (2)	0.00046 (18)	0.00314 (17)	0.00112 (17)
Br2	0.0196 (2)	0.0195 (2)	0.0182 (2)	−0.00415 (18)	0.00277 (18)	−0.00331 (18)
Br3	0.0352 (3)	0.0244 (3)	0.0403 (3)	−0.0022 (2)	0.0139 (2)	−0.0141 (2)
Br4	0.0146 (2)	0.0282 (3)	0.0343 (3)	−0.00233 (19)	0.0054 (2)	−0.0006 (2)
Br5	0.0218 (2)	0.0210 (2)	0.0195 (2)	0.00481 (18)	0.00270 (18)	0.00459 (18)
Br6	0.0251 (2)	0.0195 (2)	0.0153 (2)	−0.00074 (18)	0.00311 (18)	−0.00001 (18)
Br7	0.0199 (2)	0.0226 (2)	0.0144 (2)	−0.00124 (18)	0.00344 (17)	−0.00165 (18)
Br8	0.0162 (2)	0.0280 (3)	0.0426 (3)	0.00379 (19)	0.0092 (2)	0.0007 (2)
Br9	0.0336 (3)	0.0189 (2)	0.0348 (3)	0.0009 (2)	0.0132 (2)	0.0083 (2)
N1	0.0157 (18)	0.0190 (19)	0.0212 (19)	−0.0002 (15)	0.0014 (16)	0.0007 (17)
C1	0.010 (2)	0.025 (2)	0.016 (2)	−0.0002 (17)	0.0036 (17)	−0.0018 (18)
C2	0.0079 (19)	0.021 (2)	0.017 (2)	0.0017 (17)	0.0058 (17)	0.0010 (18)
C3	0.023 (2)	0.025 (3)	0.023 (2)	0.004 (2)	0.007 (2)	0.004 (2)
C4	0.029 (3)	0.015 (2)	0.037 (3)	0.003 (2)	0.013 (2)	0.005 (2)
C5	0.015 (2)	0.020 (2)	0.028 (2)	−0.0036 (18)	0.0064 (19)	−0.010 (2)
C6	0.015 (2)	0.024 (2)	0.018 (2)	0.0029 (18)	0.0037 (18)	−0.0017 (19)
C7	0.017 (2)	0.016 (2)	0.020 (2)	0.0037 (17)	0.0061 (18)	0.0018 (18)
C8	0.020 (2)	0.017 (2)	0.014 (2)	0.0030 (18)	0.0065 (18)	−0.0061 (18)
C9	0.017 (2)	0.015 (2)	0.015 (2)	0.0003 (17)	0.0058 (17)	−0.0042 (17)
C10	0.017 (2)	0.022 (2)	0.016 (2)	0.0059 (18)	0.0028 (18)	0.0047 (19)
C11	0.017 (2)	0.020 (2)	0.023 (2)	0.0021 (18)	0.0103 (19)	0.0045 (19)
C12	0.014 (2)	0.017 (2)	0.023 (2)	−0.0004 (17)	0.0049 (18)	−0.0039 (19)
C13	0.021 (2)	0.024 (2)	0.014 (2)	0.0036 (19)	0.0054 (18)	0.0017 (19)
C14	0.023 (2)	0.018 (2)	0.017 (2)	0.0006 (18)	0.0068 (19)	0.0034 (18)
C15	0.015 (2)	0.024 (2)	0.015 (2)	0.0011 (18)	0.0020 (17)	0.0021 (18)
C16	0.010 (2)	0.022 (2)	0.020 (2)	−0.0021 (17)	0.0047 (17)	−0.0014 (19)
C17	0.024 (2)	0.027 (3)	0.027 (2)	−0.006 (2)	0.012 (2)	−0.010 (2)
C18	0.029 (3)	0.016 (2)	0.035 (3)	−0.003 (2)	0.012 (2)	−0.006 (2)
C19	0.014 (2)	0.017 (2)	0.029 (2)	−0.0005 (17)	0.0073 (19)	0.0048 (19)
C20	0.018 (2)	0.017 (2)	0.021 (2)	0.0003 (18)	0.0030 (19)	−0.0015 (18)
C21	0.017 (2)	0.012 (2)	0.019 (2)	0.0015 (17)	0.0053 (18)	−0.0006 (17)
C22	0.015 (2)	0.014 (2)	0.016 (2)	−0.0024 (17)	0.0037 (17)	0.0048 (17)
C23	0.014 (2)	0.014 (2)	0.018 (2)	−0.0047 (16)	0.0046 (17)	0.0018 (17)
C24	0.017 (2)	0.026 (3)	0.018 (2)	−0.0038 (19)	0.0021 (18)	−0.0047 (19)
C25	0.022 (2)	0.019 (2)	0.025 (2)	−0.0045 (19)	0.011 (2)	−0.0086 (19)
C26	0.011 (2)	0.020 (2)	0.027 (2)	0.0004 (17)	0.0046 (18)	0.001 (2)
C27	0.016 (2)	0.029 (3)	0.016 (2)	−0.0005 (19)	0.0036 (18)	−0.001 (2)
C28	0.016 (2)	0.025 (2)	0.017 (2)	−0.0013 (19)	0.0049 (18)	−0.0014 (19)
C29	0.019 (2)	0.019 (2)	0.016 (2)	0.0001 (18)	0.0046 (18)	0.0028 (18)
C30	0.015 (2)	0.024 (3)	0.026 (2)	0.0026 (19)	0.0021 (19)	0.001 (2)
C31	0.025 (2)	0.027 (3)	0.015 (2)	−0.005 (2)	0.0020 (19)	0.001 (2)
C32	0.033 (3)	0.023 (2)	0.017 (2)	0.001 (2)	0.003 (2)	−0.001 (2)
C33	0.028 (3)	0.025 (3)	0.016 (2)	0.007 (2)	0.004 (2)	−0.001 (2)
C34	0.017 (2)	0.028 (3)	0.017 (2)	0.0012 (19)	0.0031 (18)	−0.0010 (19)
C35	0.024 (3)	0.023 (3)	0.038 (3)	−0.002 (2)	0.004 (2)	0.005 (2)
C36	0.043 (3)	0.023 (3)	0.028 (3)	0.001 (2)	0.011 (2)	−0.004 (2)
C37	0.025 (3)	0.032 (3)	0.027 (3)	0.006 (2)	−0.001 (2)	0.007 (2)

### Geometric parameters (Å, °)

**Table:**

Sn1—C1	2.163 (4)	C15—H15A	0.9900
Sn1—C8	2.167 (4)	C15—H15B	0.9900
Sn1—Br1	2.6203 (5)	C16—C17	1.394 (6)
Sn1—Br2	2.5051 (5)	C16—C21	1.398 (6)
Sn1—Br6	3.0470 (5)	C17—C18	1.378 (7)
Sn2—C15	2.165 (4)	C17—H17	0.9500
Sn2—C22	2.160 (4)	C18—C19	1.375 (6)
Sn2—Br6	2.9818 (5)	C18—H18	0.9500
Sn2—Br5	2.5057 (5)	C19—C20	1.375 (6)
Sn2—Br7	2.6423 (5)	C20—C21	1.383 (6)
Br3—C5	1.906 (4)	C20—H20	0.9500
Br4—C12	1.908 (4)	C21—H21	0.9500
Br8—C26	1.911 (4)	C22—C23	1.494 (6)
Br9—C19	1.908 (4)	C22—H22A	0.9900
N1—C36	1.502 (6)	C22—H22B	0.9900
N1—C29	1.502 (6)	C23—C24	1.381 (6)
N1—C35	1.505 (6)	C23—C28	1.387 (6)
N1—C37	1.509 (6)	C24—C25	1.394 (6)
C1—C2	1.483 (6)	C24—H24	0.9500
C1—H1A	0.9900	C25—C26	1.358 (6)
C1—H1B	0.9900	C25—H25	0.9500
C2—C3	1.391 (6)	C26—C27	1.385 (6)
C2—C7	1.393 (6)	C27—C28	1.383 (6)
C3—C4	1.380 (7)	C27—H27	0.9500
C3—H3	0.9500	C28—H28	0.9500
C4—C5	1.374 (7)	C29—C34	1.389 (6)
C4—H4	0.9500	C29—C30	1.391 (6)
C5—C6	1.371 (6)	C30—C31	1.380 (7)
C6—C7	1.392 (6)	C30—H30	0.9500
C6—H6	0.9500	C31—C32	1.384 (7)
C7—H7	0.9500	C31—H31	0.9500
C8—C9	1.493 (6)	C32—C33	1.384 (7)
C8—H8A	0.9900	C32—H32	0.9500
C8—H8B	0.9900	C33—C34	1.388 (7)
C9—C14	1.383 (6)	C33—H33	0.9500
C9—C10	1.402 (6)	C34—H34	0.9500
C10—C11	1.391 (6)	C35—H35A	0.9800
C10—H10	0.9500	C35—H35B	0.9800
C11—C12	1.375 (6)	C35—H35C	0.9800
C11—H11	0.9500	C36—H36A	0.9800
C12—C13	1.380 (6)	C36—H36B	0.9800
C13—C14	1.387 (6)	C36—H36C	0.9800
C13—H13	0.9500	C37—H37A	0.9800
C14—H14	0.9500	C37—H37B	0.9800
C15—C16	1.496 (6)	C37—H37C	0.9800
			
C1—Sn1—C8	141.50 (16)	H15A—C15—H15B	107.9
C1—Sn1—Br2	107.72 (12)	C17—C16—C21	118.5 (4)
C8—Sn1—Br2	107.69 (12)	C17—C16—C15	120.0 (4)
C1—Sn1—Br1	94.91 (12)	C21—C16—C15	121.5 (4)
C8—Sn1—Br1	95.68 (12)	C18—C17—C16	120.9 (4)
Br2—Sn1—Br1	96.480 (17)	C18—C17—H17	119.5
C1—Sn1—Br6	83.61 (12)	C16—C17—H17	119.5
C8—Sn1—Br6	84.91 (12)	C19—C18—C17	119.1 (4)
Br2—Sn1—Br6	84.913 (16)	C19—C18—H18	120.5
Br1—Sn1—Br6	178.233 (17)	C17—C18—H18	120.5
C15—Sn2—C22	138.41 (16)	C20—C19—C18	121.7 (4)
C22—Sn2—Br5	109.48 (11)	C20—C19—Br9	119.3 (3)
C15—Sn2—Br5	110.23 (12)	C18—C19—Br9	118.9 (3)
C22—Sn2—Br7	94.42 (12)	C19—C20—C21	119.1 (4)
C15—Sn2—Br7	93.82 (12)	C19—C20—H20	120.5
Br5—Sn2—Br7	95.245 (17)	C21—C20—H20	120.5
C22—Sn2—Br6	86.42 (12)	C20—C21—C16	120.6 (4)
C15—Sn2—Br6	85.60 (12)	C20—C21—H21	119.7
Br5—Sn2—Br6	84.392 (16)	C16—C21—H21	119.7
Br7—Sn2—Br6	179.163 (18)	C23—C22—Sn2	110.2 (3)
Sn2—Br6—Sn1	122.278 (17)	C23—C22—H22A	109.6
C36—N1—C29	109.2 (3)	Sn2—C22—H22A	109.6
C36—N1—C35	107.0 (4)	C23—C22—H22B	109.6
C29—N1—C35	113.2 (3)	Sn2—C22—H22B	109.6
C36—N1—C37	110.0 (4)	H22A—C22—H22B	108.1
C29—N1—C37	110.3 (4)	C24—C23—C28	118.1 (4)
C35—N1—C37	107.0 (4)	C24—C23—C22	121.1 (4)
C2—C1—Sn1	111.9 (3)	C28—C23—C22	120.8 (4)
C2—C1—H1A	109.2	C23—C24—C25	121.1 (4)
Sn1—C1—H1A	109.2	C23—C24—H24	119.5
C2—C1—H1B	109.2	C25—C24—H24	119.5
Sn1—C1—H1B	109.2	C26—C25—C24	119.2 (4)
H1A—C1—H1B	107.9	C26—C25—H25	120.4
C3—C2—C7	118.0 (4)	C24—C25—H25	120.4
C3—C2—C1	119.6 (4)	C25—C26—C27	121.7 (4)
C7—C2—C1	122.4 (4)	C25—C26—Br8	120.5 (3)
C4—C3—C2	121.2 (4)	C27—C26—Br8	117.8 (3)
C4—C3—H3	119.4	C28—C27—C26	118.2 (4)
C2—C3—H3	119.4	C28—C27—H27	120.9
C5—C4—C3	119.0 (4)	C26—C27—H27	120.9
C5—C4—H4	120.5	C27—C28—C23	121.8 (4)
C3—C4—H4	120.5	C27—C28—H28	119.1
C6—C5—C4	122.0 (4)	C23—C28—H28	119.1
C6—C5—Br3	119.0 (3)	C34—C29—C30	120.5 (4)
C4—C5—Br3	118.9 (4)	C34—C29—N1	121.6 (4)
C5—C6—C7	118.3 (4)	C30—C29—N1	117.8 (4)
C5—C6—H6	120.8	C31—C30—C29	119.8 (4)
C7—C6—H6	120.8	C31—C30—H30	120.1
C6—C7—C2	121.3 (4)	C29—C30—H30	120.1
C6—C7—H7	119.3	C30—C31—C32	120.3 (4)
C2—C7—H7	119.3	C30—C31—H31	119.8
C9—C8—Sn1	109.6 (3)	C32—C31—H31	119.8
C9—C8—H8A	109.8	C33—C32—C31	119.5 (5)
Sn1—C8—H8A	109.8	C33—C32—H32	120.2
C9—C8—H8B	109.8	C31—C32—H32	120.2
Sn1—C8—H8B	109.8	C32—C33—C34	121.1 (4)
H8A—C8—H8B	108.2	C32—C33—H33	119.5
C14—C9—C10	118.4 (4)	C34—C33—H33	119.5
C14—C9—C8	121.0 (4)	C33—C34—C29	118.7 (4)
C10—C9—C8	120.6 (4)	C33—C34—H34	120.6
C11—C10—C9	120.5 (4)	C29—C34—H34	120.6
C11—C10—H10	119.7	N1—C35—H35A	109.5
C9—C10—H10	119.7	N1—C35—H35B	109.5
C12—C11—C10	119.0 (4)	H35A—C35—H35B	109.5
C12—C11—H11	120.5	N1—C35—H35C	109.5
C10—C11—H11	120.5	H35A—C35—H35C	109.5
C11—C12—C13	121.9 (4)	H35B—C35—H35C	109.5
C11—C12—Br4	119.6 (3)	N1—C36—H36A	109.5
C13—C12—Br4	118.5 (3)	N1—C36—H36B	109.5
C12—C13—C14	118.5 (4)	H36A—C36—H36B	109.5
C12—C13—H13	120.8	N1—C36—H36C	109.5
C14—C13—H13	120.8	H36A—C36—H36C	109.5
C9—C14—C13	121.7 (4)	H36B—C36—H36C	109.5
C9—C14—H14	119.1	N1—C37—H37A	109.5
C13—C14—H14	119.1	N1—C37—H37B	109.5
C16—C15—Sn2	112.1 (3)	H37A—C37—H37B	109.5
C16—C15—H15A	109.2	N1—C37—H37C	109.5
Sn2—C15—H15A	109.2	H37A—C37—H37C	109.5
C16—C15—H15B	109.2	H37B—C37—H37C	109.5
Sn2—C15—H15B	109.2		
			
C22—Sn2—Br6—Sn1	43.80 (11)	Sn2—C15—C16—C17	85.2 (4)
C15—Sn2—Br6—Sn1	−95.34 (12)	Sn2—C15—C16—C21	−95.5 (4)
Br5—Sn2—Br6—Sn1	153.80 (2)	C21—C16—C17—C18	−1.4 (7)
C1—Sn1—Br6—Sn2	−100.97 (12)	C15—C16—C17—C18	177.9 (4)
C8—Sn1—Br6—Sn2	42.22 (12)	C16—C17—C18—C19	−0.5 (7)
Br2—Sn1—Br6—Sn2	150.53 (2)	C17—C18—C19—C20	2.2 (7)
C8—Sn1—C1—C2	177.6 (3)	C17—C18—C19—Br9	−176.1 (4)
Br2—Sn1—C1—C2	−26.4 (3)	C18—C19—C20—C21	−1.8 (7)
Br1—Sn1—C1—C2	72.0 (3)	Br9—C19—C20—C21	176.5 (3)
Br6—Sn1—C1—C2	−109.0 (3)	C19—C20—C21—C16	−0.3 (6)
Sn1—C1—C2—C3	90.6 (4)	C17—C16—C21—C20	1.8 (6)
Sn1—C1—C2—C7	−89.3 (4)	C15—C16—C21—C20	−177.5 (4)
C7—C2—C3—C4	0.2 (7)	C15—Sn2—C22—C23	169.7 (3)
C1—C2—C3—C4	−179.8 (4)	Br5—Sn2—C22—C23	7.6 (3)
C2—C3—C4—C5	0.6 (7)	Br7—Sn2—C22—C23	−89.5 (3)
C3—C4—C5—C6	−1.0 (7)	Br6—Sn2—C22—C23	90.4 (3)
C3—C4—C5—Br3	−179.6 (4)	Sn2—C22—C23—C24	−100.2 (4)
C4—C5—C6—C7	0.6 (7)	Sn2—C22—C23—C28	77.0 (4)
Br3—C5—C6—C7	179.2 (3)	C28—C23—C24—C25	0.9 (7)
C5—C6—C7—C2	0.2 (7)	C22—C23—C24—C25	178.1 (4)
C3—C2—C7—C6	−0.6 (6)	C23—C24—C25—C26	−2.0 (7)
C1—C2—C7—C6	179.4 (4)	C24—C25—C26—C27	2.1 (7)
C1—Sn1—C8—C9	174.0 (3)	C24—C25—C26—Br8	180.0 (3)
Br2—Sn1—C8—C9	17.9 (3)	C25—C26—C27—C28	−1.2 (7)
Br1—Sn1—C8—C9	−80.8 (3)	Br8—C26—C27—C28	−179.1 (3)
Br6—Sn1—C8—C9	100.9 (3)	C26—C27—C28—C23	0.1 (7)
Sn1—C8—C9—C14	89.1 (4)	C24—C23—C28—C27	0.1 (7)
Sn1—C8—C9—C10	−89.0 (4)	C22—C23—C28—C27	−177.2 (4)
C14—C9—C10—C11	−0.8 (6)	C36—N1—C29—C34	118.1 (5)
C8—C9—C10—C11	177.4 (4)	C35—N1—C29—C34	−1.1 (6)
C9—C10—C11—C12	0.3 (7)	C37—N1—C29—C34	−120.9 (4)
C10—C11—C12—C13	0.4 (7)	C36—N1—C29—C30	−62.2 (5)
C10—C11—C12—Br4	179.4 (3)	C35—N1—C29—C30	178.7 (4)
C11—C12—C13—C14	−0.6 (7)	C37—N1—C29—C30	58.8 (5)
Br4—C12—C13—C14	−179.6 (3)	C34—C29—C30—C31	−1.5 (7)
C10—C9—C14—C13	0.5 (6)	N1—C29—C30—C31	178.8 (4)
C8—C9—C14—C13	−177.7 (4)	C29—C30—C31—C32	0.6 (7)
C12—C13—C14—C9	0.2 (7)	C30—C31—C32—C33	0.9 (7)
C22—Sn2—C15—C16	171.9 (3)	C31—C32—C33—C34	−1.6 (7)
Br5—Sn2—C15—C16	−26.2 (3)	C32—C33—C34—C29	0.8 (7)
Br7—Sn2—C15—C16	70.9 (3)	C30—C29—C34—C33	0.8 (7)
Br6—Sn2—C15—C16	−108.5 (3)	N1—C29—C34—C33	−179.5 (4)

### Hydrogen-bond geometry (Å, °)

**Table:**

Cg is the centroid of the C29-benzene ring.

**Table:**

*D*—H···*A*	*D*—H	H···*A*	*D*···*A*	*D*—H···*A*
C27—H27···Cg^i^	0.95	2.58	3.378 (4)	142

Symmetry codes: (i) −*x*+1, −*y*+1, −*z*+1.
